# New Methods of Mounting Artificial Teeth

**Published:** 1880-05

**Authors:** 


### AMERICAN JOURNAL

OF

### DENTAL SCIENCE.

Vol. XIV. THIRD SERIES--MAY, 1880. No. 1.

### ARTICLE I.

*New Methods of Mounting Artificial Teeth.*—(Continued.)

[Transactions of Ohio State Dental Society.]

Dr. J. Taft.—There is another point in reference to artificial plates, to which I think too little attention is given, and that is, the adjusting of plates bearing a few teeth—partial sets of teeth—adjusting them round about the natural teeth in such a way as to afford them the greatest security from injury. I think every one of much experience will have recognized the fact that in a great majority of cases where partial sets of teeth are worn, the gums around the natural teeth, that may be standing, are more or less diseased. The margins of the gum may be irritated in two or three ways. If the plate brought up around about the tooth, is made to impinge upon it, it will invariably produce disease of that gum. Sometimes there is simply irritation; in some cases inflammation; in others hypertrophy—enlargement of the gums; some fit the plate up very close to the adjoining teeth; others are not careful about this, letting it come in contact or not; and some leave it some distance away, as it may happen, without any special design. Some again are careful to get the plate away a considerable distance from the margins of the gums, so this influence shall not be exerted upon them. I have considered it a matter of importance that the margins of the gums about such teeth should receive special attention and be properly guarded. Now the point is to properly guard the gum. If it is diseased, or if it is simply irritated, its product is vitiated and will sooner or later become injurious to the teeth and cause them to decay. We see a great many teeth decay in the vicinity of plates from these two causes, the vicious product arising from irritation at the margin of the gum and from the lodgment and decomposition of foreign substances. Now, what is the best way to arrange plates around the teeth, so as to best secure the gums and teeth, so as to avoid irritating the gums, and to prevent the lodgment of foreign material? Gentlemen who are working in laboratories and working with partial sets of teeth have their opinion. But what can we do to protect the teeth from the lodgment of foreign substances and the irritation of the gums? Of course different cases will be differently affected. Some are so susceptible to the presence of foreign substances that any one almost will produce more or less irritation. Others again—and quite a majority, perhaps, bear a plate very well without showing any signs of irritation, especially if the plate is of proper material.

Dr. A. Berry.—I think these questions of Dr. Taft's are very pertinent and very important, and pertain to matters of great consequence to persons wearing artificial teeth. This matter of artificial plates has for the last few years, by a great many of the best operators in our profession, been entirely too much ignored. There are a great many men who are splendid operators in the way of filling teeth, but when it comes to the matter of plates their consciences are not clear. That part of the practice is too much neglected by the profession. I know there is not the money in mechanical dentistry that there is in operative, and not so much honor; but that does not exhonerate us from doing the best we can for those who wear artificial teeth.

Now in reference to this matter of plates impinging on the gums, and the lodgment of foreign matter about the natural teeth, much may be said. The plates may be brought closely up to the natural teeth when the parts are all healthy, and upon fitting them closely you may think it is all right and nice, but if you make a nice adaptation to the necks of teeth where there is already diseased action, or a tendency to it, as may be determined in most cases by close inspection, you invariably cause diseased action to follow. In many cases when plates are made to fit closely and nicely—and we used to do so to a greater extent when we made gold plates than now—they produced disease and disorder, leading patients to complain, and often a little must be cut away to give relief. A great deal of discrimination is necessary to know what to do in the outset without waiting for consequences. More attention and study should be given to this subject. The health and comfort of the patient depends very much on these matters, over which the dentist has control; and he should see to it that the most perfect plates of the best manufacture, are so skillfully set and arranged as to give the most comfort and satisfaction to the wearer.

Dr. F. H. Rehwinkel.—There is one point in this matter to which I wish to refer; that is, that the material in some cases has something to do with it, and on that ground, I endeavor as much as possible to have persons use nothing but gold for partial sets of teeth. I exhaust every argument I can use on the subject, before consenting to insert rubber for partial sets.

Now I have never been able to determine—because I have not made experiments with it systematically—whether one method is better than another, and whether we should let the plate go close to the margin of the gum on the lingual surface of the teeth, as Dr. Berry said, must be determined by the condition of the mucous membrane of the mouth. If, for instance, there is a flabby condition the gums, which inclines to inflammation, I apprehend a plate were in that case fitted closely to the gums, you would have trouble, unless you take the precaution to impress upon the patient as strongly as you can, never to wear any kind of plates at night. I know a great many dentists differ with me in this matter; but I know it is one of the greatest things to avert disease in some mouths. Those who have worn artificial teeth themselves and have not observed that caution may have become used to it. But if you consider for a moment that for the space of from twelve to eighteen hours—say fourteen hours—you have a strange substance in the mouth, over the mucous membrane, interfering with the circulation, you must be convinced that unless you can give those parts time to rest, and allow the capillaries to become filled again, and the natural channels of circulation to be restored, you will have trouble.

This is one of the points I insist upon; and always advise removal of the plates at night. I have seen cases where this condition of disease existed in the mouth, and upon asking the patient if the plates were not worn at night, found this to be the case; when upon following my advice, to discontinue doing so, the trouble which existed has been very much alleviated, if not entirely removed,

It is now my turn to ask questions. I would ask Dr. Taft what has become of the method called "Hall's method." You will remember that the subject was introduced some years since, and it was claimed that it was done by cutting dovetailed grooves in the teeth. I have heard nothing of it lately. I believe they were promised as soon as he could make arrangements with Dr. S. S. White for the purpose of getting up some patterns.

Dr. Taft.—There were some good features about them; some principles which, if carried out, would be very good. He had some difficulty in getting the teeth made, and I presume that was the chief embarassment about it. White, and indeed all the manufacturers, have expended large amounts of money and labor in molds, etc., for the manufacture of teeth, and might not have felt like venturing into an enterprise calling for a large outlay in addition to that already incurred, as it would have required new molds, as I understand it.

Dr. Rehwinkel.—The difficulty which occurred to me in regard to them was the length of the teeth. It did not seem possible to me to have the teeth short enough to afford room for these grooves.

Dr. J. Taft.—I also thought of that at the time, but possibly that might have been overcome if he had had the means of carrying out his idea, but I presume Dr. Hall had not.

Now, in reference to plates about natural teeth. The points have been pretty well presented here, and I fully endorse what has been said about cutting the plate back from the teeth. I have not, for a great while at least, been in the habit of fitting plates closely around the teeth. The argument is sometimes used that you should fit it just as closely around the necks of the teeth as possible, and that then the gum is less likely to be affected. But I have noticed that sometimes even in such cases the difficulty would ensue, that the gum would get out, and not only would the tooth but the alveolus as well would be injured. So that danger is liable to occur in two directions, decay and removal of the support of the teeth. As Dr. Berry said, it is necessary to discriminate in the different cases, and when there is a large amount of soft tissue, employ the means adapted to the case. Then the edge of the plates about the margin should be either further away from the teeth or fit them perfectly. If the integuments are not thick it might be made closer than in the other case. But I don't see what is gained by forcing the plate up close to the teeth. Get away as far as possible from the margin of the gum and then make such an adaptation of the edge of the plate as will not impinge upon mucous membrane enough to inflame it. That ought to be regarded in every case and arranged so that it will rest uniformly upon the gum and not make a pressure anywhere so as to induce irritation or inflammation. So, my idea for years has been, in adjusting a plate, to get it as far away from the natural teeth as possible—the farther the better. I regard this as an important point, because the natural teeth are better than artificial, and where there are only two or three natural teeth it is very important to save them.

Dr. W. M. Herriott thought the plan used by Dr. Carter probably a good thing. Around the air chamber he had a piece of gold, aluminum, platinum, or something of the kind coming in such a way as that it could only force down, being held there in perfect condition, allowing the roof of the mouth to be kept in better condition, because of that being a conductor of heat and cold.

He concurred with Dr. Rehwinkel in reference to partial sets of teeth; that is, that dentists should insist upon patients removing their artificial teeth at night.

Dr. H. A. Smith said that spring plates, to which reference had been made, were not usually satisfactory after being worn for any considerable length of time. The teeth were soon forced out of position by the pressure upon them, and tne plate would drop. I recently saw a plate made in Germany, in which wooden pegs had been inserted after the plate had become loosened, so as to form bearings upon the natual teeth, in order to steady it in its place. These wooden pegs had to be renewed from time to time. I have seen the same method resorted to where gald plates were worn. Tubes of the metal to receive the wooden bearings were soldered on the plate. By the judicious placing of these bearings, many loose plates may be rendered comfortable and serviceable.

Dr. J. W. Lyder mentioned an operation in which a central incisor was made to give good satisfaction by soldering a very little flange upon the inner surface of the plate. It was a single porcelain tooth, the articulation being made as in cases where the operator was going to pack with gold packing.

Dr. J. Taft thought that by combining the plans spoken of, great advantages might be secured. In illustration of this, he said: If for example there were two or three good roots, and two or three teeth to be replaced, where teeth had been entirely removed, make a narrow plate which would occupy all these spaces, and where the roots were. Attach the plate to the roots by closely fitting collars. Solder into the plate; then when the teeth are attached, press the piece to its place on the roots. This narrow plate could be put into the space desired, and so much of the roots utilized as requisite, and the plate be finished up in a proper manner.

He stated that he had seen plates pivoted on roots worn for many years, and the question was whether narrow plates, properly adjusted in the way mentioned, could not be made so as to stand very firm and much better than the ordinary clasping, and better than plates pivoted into the roots, because of their having more bearing.

One thing referred to by Dr. Butler, he considered very important; that was not to destroy the pulp in roots where it was alive, but save it alive and treat it as in cases of exposed pulp.

Dr. Corydon Palmer—I would like to make a remark upon the adjusting of plates next to the teeth. It has been said, very truly, that it was best to keep as far away from the teeth as possible, and I admit that, but I would like something definite. How far can you get away? What has been said is hardly definite enough on this point. Why don't you give some plan or system? You can get mine. If I make a plate for two lateral incisors, supposing it to be a metal plate, I would, after securing sufficient size for the plate, which would contain an air chamber, extend an arm to each lateral incisor, make the chamber shallow and leave as much of the other parts exposed as possible to give strength.

Dr. W. M. Herriott.—If I remember distinctly, Dr. Rehwinkel, you undertook some years ago to see what could be accomplished in cutting away an upper plate, to see if it would sustain the teeth. What was the result?

Dr. Rehwinkel.—The result was satisfactory; and, I think, would be to anyone else, who had worn artificial plates before, and had gained some experience. You all know how troublesome it is sometimes, to convince patients that they have a properly fitting plate. Their want of experience in exhausting the air, and then their want of confidence in their own ability to manage the plate, and the constant fear that the plate may drop down into the mouth at an inopportune mement, destroy, for the time being, all the sense of comfort, and causes the patient often to become discouraged. If this occurs with the ordinary plates—with an air chamber even—you can imagine how improbable it would seem that such patients would be satisfied with a plate, of which the palatal portion is entirely cut away, and only enough of the plate left to cover the alveolar ridge. The plates are in shape like the gold plates made for the upper maxillary some thirty years ago, when spiral springs were relied upon. The method Dr. Palmer just referred to—leaving as little of the plate as possible around the margin of the air cavity—would be a great advantage, because the margins of the gums on the lingual surface of the teeth will be more thoroughly relieved of pressure and interference.

Dr. L. Buffett.—We all know the relation which the gum tissue has to the alveolar process of the teeth, that the gum tissue extends above the alveolar process. Now if the plate is brought in contact with the teeth so that there is pressure upon this gum tissue above the alveolus, you will have inflammation. If it stops at the margin you will have absorption. And I make this statement on the ground that there is always inflammatory action on the margin or under every plate. So if the plate does not extend to the edge of the alveolar process you will not have recession; if there is irritation at the edge of the process you will have absorption or perhaps have hypertrophy of the gums. —*Transactions of Ohio State Dental Society.*

